# FOXA1 can be modulated by HDAC3 in the progression of epithelial ovarian carcinoma

**DOI:** 10.1186/s12967-021-03224-3

**Published:** 2022-01-06

**Authors:** Tong Lou, Chongdong Liu, Hong Qu, Zhiqiang Zhang, Shuzhen Wang, Huiyu Zhuang

**Affiliations:** Department of Obstetrics and Gynecology, Beijing Chaoyang Hospital, Capital Medical University, No.8, North Road of Workers Stadium, Chaoyang District, Beijing, 100020 China

**Keywords:** Epithelial ovarian carcinoma, HDAC3, FOXA1, Progression, Wnt/β-catenin signaling pathway

## Abstract

FOXA1 is associated with malignant tumors, but the function of FOXA1 in EOC is unclear. HDAC3 can influence the proliferation, migration and invasion ability of EOC. In this study, we wanted to explore the function of FOXA1 in ovarian cancer and the relationship between HDAC3 and FOXA1.The expression of HDAC3 and FOXA1 was detected by immunohistochemical staining of primary lesions from 127 epithelial ovarian carcinoma patients. A proliferation assay, a Transwell assay, an apoptosis assay and animal experiments were used to assess the proliferation, invasion and apoptosis abilities of ovarian cancer cells before and after transfection with FOXA1. The relevance of the in vitro findings was confirmed in xenografts. The H-scores for FOXA1 and HDAC3 staining in FIGO stage III-IV were noticeably higher and predicted adverse clinical outcomes in patients with ovarian cancer. The expression level of HDAC3 was significantly correlated with the expression level of FOXA1. Invasion, proliferation and apoptosis capacity and tumor formation were decreased in the FOXA1-knockdown cells. Experiments in xenografts confirmed that HDAC3 mediated tumor formation. In conclusion, FOXA1 can be modulated by HDAC3 through the Wnt/β-catenin signaling pathway, and FOXA1 plays essential roles in the proliferation, apoptosis and invasion of EOC cell lines and xenograft experiments.

## Introduction

Epithelial ovarian carcinoma (EOC) is one of the leading causes of cancer-related death among women, accounting for 195,000 new cases and 185,000 deaths annually worldwide [1]. Due to its asymptomatic nature and lack of effective screening tests, the majority of patients are diagnosed at advanced stages [2]. Patients with a poor prognosis may have early tumor metastasis, drug resistance and recurrence [3, 4]. Thus, evaluating the mechanisms of EOCs at the molecular level is important to help early diagnosis and treatment of ovarian cancer and to improve the prognosis of patients with this disease.

Histone deacetylase 3 (HDAC3) is a member of the histone deacetylase family that is associated with cellular physiological functions, such as signal transduction, cell cycle, proliferation, apoptosis, and cardiac development. HDAC3 plays an important role in the progression of malignant tumors, especially in proliferation [5], apoptosis [6], metastasis [7], angiogenesis [8] and anticancer drug resistance [9]. In our previous study, we discovered that HDAC3 can influence the proliferation, migration and invasion ability of EOC [10]. However, tumor progression is a complex pathophysiological process, and HDAC3 alone cannot control biological behavior. Further studies are necessary to investigate the HDAC3-related proteins.

The Fox protein family is a family of transcription factors that exist widely from yeast to humans. It has a DNA-binding region composed of three α helices and two sides of β-pleated sheets, which is highly conserved and known as the foxhead frame structure. FOXA is a subtype of the Fox protein family and has three subtypes: FOXA1, FOXA2 and FOXA3. FOXA1 was first identified as a transcription factor in 1989 and was then found to play an important role in the development and differentiation of various tissues [11–14]. FOXA1 is abnormally expressed in multiple malignant tumors. It is related to poor prognosis and influences proliferation capacity, apoptosis capacity, and invasion capacity [15, 16]. In our previous study, the results of gene chip detection demonstrated that decreasing the expression level of HDAC3 inhibits FOXA1 expression (unpublished data). Therefore, we hypothesized that HDAC3 may have a relationship with FOXA1.

The Wnt/β-catenin signaling pathway is an important pathway. It is closely related to embryo development, tissue self-renewal and various diseases. The Wnt/β-catenin signaling pathway can regulate cell proliferation, apoptosis and invasion [17–19]. It has also been reported to be abnormally activated in various types of cancers, including ovarian cancer [20, 21]. HDAC3 can regulate the Wnt/β-catenin signaling pathway [22],in lung cancer study, they found that loss the expression of HDAC3 dierupted the expression of multiple Wnt ligands, resulting in a reduction of canonical Wnt activity.

In the present study, we explored the relationship of HDAC3 and FOXA1 in EOC and the function of FOXA1 in EOC progression through in vitro and in vivo assays.

## Material and methods

### Clinical data and tissue specimens

The medical records of all consecutive patients diagnosed and treated for EOCs from January, 2010 to December, 2018 in Beijing Chao-Yang hospital. Patients who had the primary malignant tumor in another part of body were not included in this study. Patients without complete surgery and pathology reports or who were lost to follow-up within one month after the initial surgery were also excluded from this study. Patient information, including demographic and pathological characteristics, and disease status at the last contact, were collected and evaluated. Additionally, paraffin-embedded tissues from the enrolled patients were obtained from the pathology center. In our study, tumor staging was based on the 2015 International Federation of Gynecology and Obstetrics (FIGO) system [23]. The progression-free survival (PFS) was defined as the date of surgery to the date of recurrence; patients who hadn’t recurred at their last visit were censored. The overall survival (OS) time was defined as the date of surgery to the date of patient death because of the disease, and patients died due to other conditions, disease and the patients who survived at their last visit were censored. This study was approved by the ethics committees at the Beijing Chao-Yang Hospital, and the patients involved in this study gave their informed consent.

### Tissue microarray (TMA)

TMAs were executed by matched primary tumor lesions. Two gynecological pathologists independently reexamined the pathology slides and ensure EOC diagnosis, marking the accurate the localization of the malignant lesions on the paraffin-embedded tissue samples. Round tissue samples with a diameter of 1 mm were obtained from the tumor located in the donor block using a manual tissue array instrument (TMArrayer), transferred into the TMA block. Sequential sections were cut from the paraffin-embedded TMA blocks at a thickness 4 um and placed on blank slides.

### Immunohistochemical (IHC) staining

IHC staining was performed as previously described [10]. Briefly, baking the sections in 70 ℃ for 60 min, then deparaffinized and rehydrated sections in the dimethylbenzene and graded ethanol. Using the 3% hydrogen peroxide to block endogenous peroxidase activity. Ten percent goat serum was treated at room temperature for 1 h. The sections were incubated with the primary antibody (Rabbit anti-HDAC3, 1:20 and mouse anti-FOXA1, 1:100) at 4 ℃ over night. After recovering to room temperature, adding the horseradish peroxidase-conjugated goat anti-rabbit/mouse antibody for 1 h, tissue sections were subjected to diaminobenzidine staining for color development. Subsequently, sections were subjected to hematoxylin counterstaining and dehydration, and then sealed with neutral resin.

### Evaluation of the IHC staining of the TMA

Utilizing a digital pathological section scanner (Pannoramic MIDI/P250) to capture images of the TMA slides, then displaying the imaging at 400× magnification by panoramic Viewer 1.15.4 software. Two gynecologists independently evaluated the images and they didn’t know the clinical data. A histochemistry score (H-score) based on a combination of the percentage of stained cells and staining intensity was calculated for the semiquantitative analysis. H-score = Σ(percentage [0–100%] × intensity [1–3]) = (percentage of cells with weak intensity × 1) + (percentage of cells with moderate intensity × 2) + (percentage of cells with strong intensity × 3) [24]. The relationship between HDAC3 and FOXA1 was analyzed, in addition, the relationship between them and the patients’ prognosis was also analyzed. We dividwed the patients into high expression group and low expression group according to the median of HDAC3 or FOXA1 H-score.

### Western blotting analysis

Using RIPA buffer to collect the total protein. Ten percent SDS-PAGE gels was performed and loading 30ug protein onto it. Then transferring the protein to a polyvinylidene difluoride (PVDF) membranes (Millipore) through 100 mV for 90 min. All membranes were treated with 0.5% BSA for 2 h and then incubated with 1:1000 dilution of rabbit anti-HDAC3 monoclonal antibody (Abcam), a 1:1000 dilution of mouse anti-FOXA1 monoclonal antibody (Abcam), 1:1000 dilution of rabbit anti-β-catenin monoclonal antibody (Cell Signaling Technology), 1:1000 dilution of rabbit anti-MMP2 monoclonal antibody (Cell Signaling Technology), 1:1000 dilution of rabbit anti-Cyclin D1 monoclonal antibody (Cell Signaling Technology), and 1:2000 dilution of rabbit anti-GAPDH monoclonal antibody (abcam) in PBS containing 0.1% Tween-20 and 5% bovine serum (BSA) overnight at 4℃. After incubation with the secondary antibody at 1:4000 dilution (Golden Bridge International, Inc., Beijing, China) for 1 h at room temperature, the blots were detected by the enhanced chemiluminescence (ECL) substrate kit (Thermo USA). The experiments were repeated three times.

### Quantitative real-time polymerase chain reaction (qRT-PCR)

Total RNA was isolated from ovarian cell lines or tissues by the TRIzol reagent (Invitrogen, Carlsbad, CA) according to the manufacturer's instructions. cDNA of each sample was reverse transcribed by approximately 2 μg of RNA. Synthesized cDNA was amplified on the ABI Biosystems 7500 Fast Real-Time PCR System with SYBR Green I Master. The cycling conditions were as follows: 95 °C for 30 s followed by 40 cycles of 95 °C for 5 s and 60 °C for 34 s for telomere PCR. The experiments were repeated three times with triplicates of each sample. The expression levels of HDAC3 and HE4 were calculated by the 2−ΔCt method. This experiment was repeated three times independently. Primer sequences were as follows: HDAC3: F: 5′-GCAAGGCTTCACCAAGAGTCT-3′, R: 5′-AGATGCGCCTGTGTAACGC-3’; FOXA1: F: 5′-GCAATACTCGCCTTACGGCT-3′, R: 5′-TACACACCTTGGTAGTACGCC-3’; and GAPDH: F: 5′-GGAGCGAGATCCCTCCAAAAT-3′, R: 5′-GGCTGTTGTCATACTTCTCATGG-3’.

### Cell culture

The human ovarian cancer cell lines (OVCAR3, A2780, SKOV3 and ES-2) were purchased from the Peking Union Medical College Cell Resource Center (Beijing, China). The OC cell line A2780 was kindly provided by Dr. Deng (Tsinghua University). A2780, OVCAR3 and SKOV3 cells were cultures in complete medium RPMI 1640 medium (Corning) containing 10% heat-inactivated FBS (Gibco), penicillin (100U/ml)/streptomycin(100ug/mL). ES-2 was cultured in McCoy's 5A Medium (Gibco) with 10% heat-inactivated FBS (Gibco), penicillin (100U/ml)/streptomycin(100ug/mL). Incubating in a humidified incubator at 37 °C under 5% CO2 in air.

### Coimmunoprecipitation

OC cells was treated with ice-cold RIPA buffer (1 ml) and incubation on ice for 30 min. The cells suspension was centrifuged at 12,000 g for 30 min at 4 °C, then discarded the supernatant and collected supernatant fractions. Anti-FOXA1 antibody (10 μl) (Abcam, rabbit monoclonal) or anti-HDAC3 antibody (CST, mouse monoclonal) was added in the supernatant fractions overnight at 4 °C. The mixture was treated with Protein A/G PLUS-Agarose (20 μl; Santa Cruz) and incubation them on the rocker platform overnight at 4 °C. The negative control was 10ul anti-Rabbit IgG (abcam). Western blot was used to analyze the immunoprecipitates with FOXA1 monoclonal (Abcam, Rabbit) and HDAC3 monoclonal (Abcam, Mouse) antibodies. ECL reagent (Amersham ECL Prime detection) was used to visualize the proteins.

### Transfection

As our primary experiment [10], SKOV3 and ES-2 were used to do the cell experiment. The lentiviral named Lv-HDAC3-shRNA and Lv-FOXA1-shRNA was purchased for knockdown the expression of HDAC3 and FOXA1, respectively. The lentiviral named Lv-HDAC3 and Lv-FOXA1 was purchased for increasing the expression of HDAC3 and FOXA1, respectively. The cells were seeded in 24-well plates with the density of 5 × 10^4^ cells/well. After 72 h of infection, lentiviral vectors encoded enhanced green fluorescent protein (eGFP) and infected cells can be observed through fluorescence microscopy. The fluorescence intensity represented transduction efficiency and eGFP expression. Cells with 80%-90% infection efficiency and better cellular growth behavior were chosen and expanded via puromycin treatment. The efficiency of the infection was confirmed by western blot and reverse transcription PCR (RT-PCR).

### Transwell invasion assay

Transwell inserts (8um pore size) were used for invasion assays and were placed in 24-well plates. Matrigel was melt before the experiment and diluted with serum-free medium at 1:20. Forty microliter Matrigel was added in the upper chambers and put the plates in a humidified incubator at 37 °C. Cells were resuspended in serum-free medium in the upper chamber at 4*10^4^ cells/well. Medium containing 10% FBS was added in the lower chamber. Culturing 16 h in humidified incubator, the medium was discarded and the migration cells was fixed by 4% paraformaldehyde for 15 min. Then the membrane was washed 3 times with PBS and stained with 0.1% crystal violet for 20 min after drying. The stained cells were counted and observed in the bright field of a microscope. Positive cells were numbered at least 5 random microscopic fields and the statistical analysis was performed.

### Cell colony forming experiment

The cells in the log phase were spread into a 6-well plate with 1,000 cells per well, culture in a CO2 cell incubator for 14 days, discard the medium, stain with 0.01% crystal violet, and count the colonies under a microscope to calculate the formation of cell colonies rate. Number of colonies = average number of cell colonies in N wells, colony formation rate% = number of colonies/total number of cultured cells × 100%.

### 5-Ethynyl-2'-deoxyuridine (EDU) assay

EdU assay (RIBOBio Co, Guangzhou, China) was used to measure cells’ abilities to proliferate. After incubation with EdU for 1 h, the cells were fixed with 4% paraformaldehyde and permeabilized with 0.5% Triton X-100. Then, the Apollo reaction cocktail (reaction buffer and Apollo® 567 fluorescence) was added to medium for another 30 min in the dark. After washed with PBS for three times, and the nuclei were stained with Hoechst 33,342 and immediately viewed under fluorescence microscopy. Cell proliferation ratios were calculated using the formulation of (Edu-positive cells/Hoechst-stained cells) × 100%. The number of EdU-positive cells were calculated by counting at least three random separate fields.

### Apoptosis assay

The assay was performed as instruction of the PE Annexin V apoptosis detection kit I (BD Pharmingen). In brief, cells in the logarithmic growth phase were washed once with PBS, then EDTA-free trypsin was added, and all the target cells attached and suspended were collected by centrifugation at 1000 rpm for 5 min. Wash cells twice with cold PBS and then resuspend cells in 1X Binding Buffer at a concentration of 1 × 10^6 cells/ml. Transfer 100 μl of the solution (1 × 10^5 cells) to a 5 ml culture tube. Add 5 μl of PE Annexin V and 5 μl 7-AAD. Gently vortex the cells and incubate for 15 min at RT (25 °C) in the dark. Add 400 μl of 1X Binding Buffer to each tube. Analyze by flow cytometry within 1 h.

### Xenograft experiments

All procedures performed in animal studies were approved by the Animal Research Ethics Committee of Capital Medical University. ES-2 was fast growing and in good condition, so it was used to do the xenograft experiment. Female BALB/c nu/nu mice (4–6 weeks old; Shanghai Institute of Material Medicine, Chinese Academy of Science) were raised in specific pathogen-free conditions at 22 °C and 55% humidity. Cells at the exponential phase of growth were digested by 0.25% trypsin and resuspended in PBS.

In the node mouse tumorigenicity, forty female nude mice were randomly divided into four groups (10 mice per group). 5*10^6^ cells/200ul were subcutaneously injected into the right flank of each nude mice. The tumor width and length were measured every 5 days. The formula of formula (width)^2^ × length/2 was calculated the tumor volumes. The nude mice were sacrificed via broking the neck without any anesthetic inhalation after 20 days of observation. The tumors were isolated, fixed with 10% formalin, and embedded in paraffin for further pathological analyses.

In mouse tail-vein injection experiments, ten female nude mice were randomly divided into two groups (Mock and HDAC3-overexpression group), A volume of 200ul (1 × 10^6^) was injected into the tail-vein of nu/nu mice and the mice were observed for 21 days. The mice were then killed humanely by broking the neck without any anesthetic.

In peritoneal metastasis assays, ten female nude mice were randomly divided into two groups (Mock and HDAC3-overexpression group). Cells (5 × 10^6^ cells/200ul PBS) were injected intraperitoneally for peritoneal metastatic formation and the mice were observed for 21 days. The mice were then killed humanely by broking the neck without any anesthetic.

An autopsy was performed, the lungs and the peritoneal were examined for tumors separately. The tissues were dehydrated, processed, and embedded in paraffin wax and stained with haematoxylin and eosin (HE).

### Statistical analysis

Statistical analyses were performed using the SPSS 22.0 statistical package. The quantitative data are presented as mean ± SD and were analyzed using ANOVA or two tailed Student’s t-tests. The Wilcoxon signed rank test was used to compare the HDAC3 and FOXA1 expression in the same patients. The two-sample rank sum test was used to analyze the relationship between HDAC3 and FOXA1expression and the prognosis. The Kaplan–Meier method was used for a univariate analysis of disease-free survival (DFS) and overall survival (OS). Different survival curves were compared using the log-rank test. The Cox proportional hazard model was utilized to evaluate all parameters that were significant in univariate analyses. The multivariate adjusted odds ratios (ORs) and 95% confidence intervals (CIs) are expressed. A two-sided P-value < 0.05 was considered statistically significant [25].

## Results

### Expression of HDAC3 and FOXA1 in human OCs tissues

One hundred and twenty-seven patients who were diagnosed with OC were selected for the present study. Table 1 shows the clinicopathological characteristics of the OC patients. The mean age at first diagnosis was 57 (range 16–87) years. Based on the FIGO 2015 staging system, 35 patients had stage I tumors, 9 patients were in stage II, and 69 and 14 patients had stage III and stage IV tumors, respectively. There were 127 ovarian primary tumor lesions in the TMAs, including 90 serous patients (70.9%), 7 mucinous patients (5.5%), 21 clear cell patients (16.5%) and 9 endometrioid patients (7.1%). Among those patients, 47 patients (37%) had lymphatic metastasis.

**Table 1:** Clinicopathological characteristics of the patients with EOC

	Mean or number	Range or percentage
Age at diagnosis (years)	57 ± 11.85	16–87
Menopause status		
Pre-menopause	33	26%
Post-menopause	94	74%
Family history of cancer		
Yes	7	5.5%
No	120	94.5%
Preoperative CA125 level (IU/ml)	1254.51 ± 2579.66	9.53–17,980
FIGO stage		
Stage I	35	27.6%
Stage II	9	7.1%
Stage III	69	54.3%
Stage IV	14	11.0%
Pathological pattern		
Serous	90	70.9%
Mucinous	7	5.5%
Clear cell	21	16.5%
Endometrioid	9	7.1%
Lymphatic metastasis		
Yes	47	37.0%
No	80	63.0%
Current status		
NED	65	51.2%
AWD	19	15.0%
DOD	43	33.9%
PFS (months)	32.598 ± 23.0	4–109
OS (months)	40.448 ± 24.5	4–109

*FIGO* International Federation of Gynecology and Obstetrics, *NED* no evidence of disease, *AWD* alive with disease, *DOD* die of disease

**Fig. 1:**
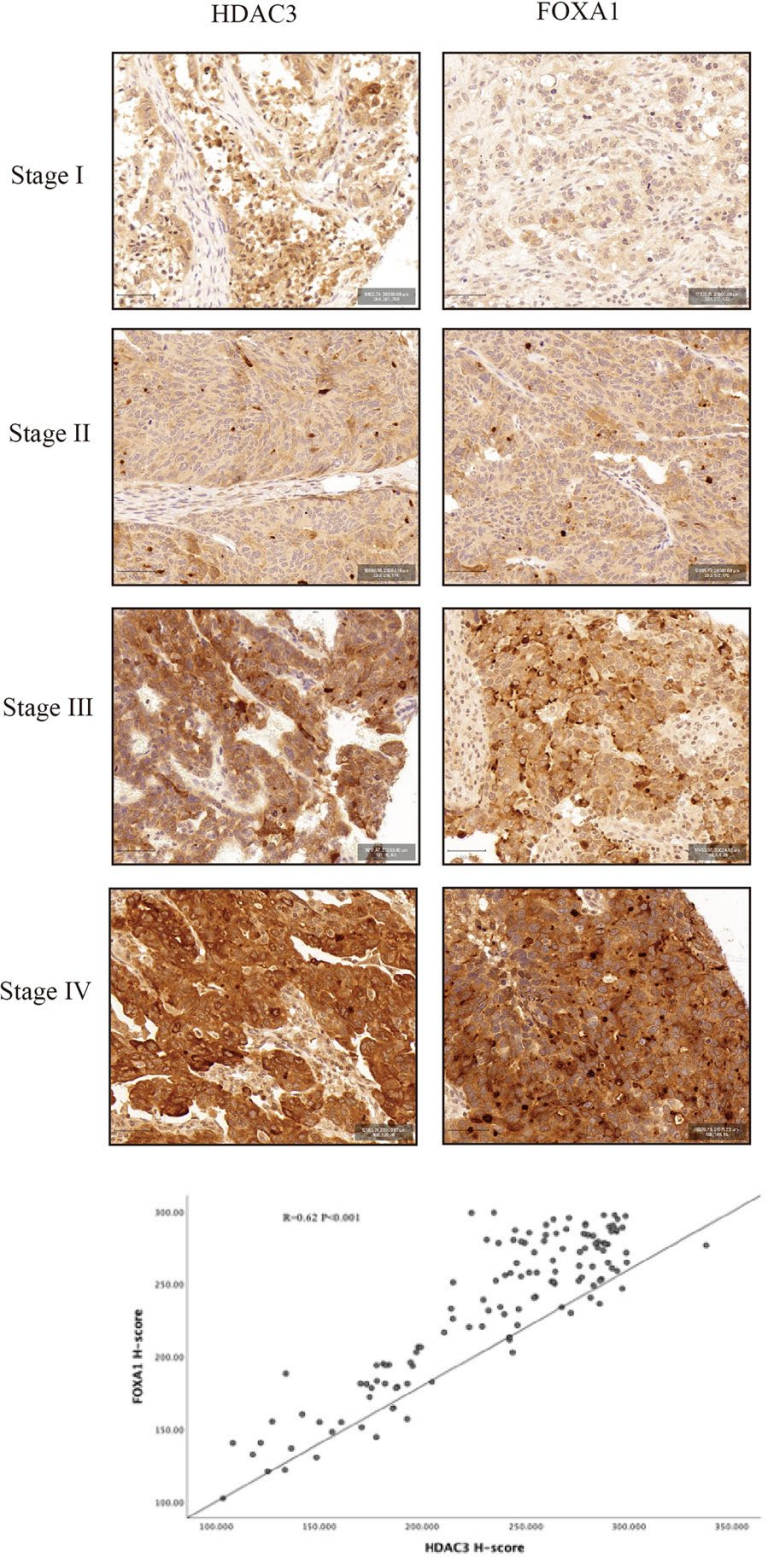
Expression of HDAC3 and FOXA1 in human OC tissues. HDAC3 and FOXA1 were detected through staining in EOCs at different stages. The expression level of HDAC3 was significantly correlated with the expression level of FOXA1 (r = 0.595, P = 0.000)

The mean H-score of HDAC3 in EOCs was 213.05 ± 51.79, and the mean H-score of FOXA1 in EOCs was 198.49 ± 57.06. In Table 2, there was no significant difference among all pathological types. The expression of HDAC3 and FOXA1 was significantly elevated in FIGO stages III-IV (HDAC3: p = 0.007, FOXA1: p < 0.001). The expression levels of HDAC3 and FOXA1 were significantly increased in ovarian cancer recurrence (HDAC3: p < 0.001, FOXA1: p = 0.002) and predicted adverse clinical outcomes in patients with ovarian cancer (HDAC3: p < 0.001, FOXA1: p < 0.001). In addition, the expression level of HDAC3 was significantly correlated with the expression level of FOXA1 (r = 0.595, P < 0.001, Fig. 1).

**Table 2:** Association between HDAC3 and FOXA1 expression and pathological features

Features	Cases	HDAC3 H-score	P value	FOXA1 H-score	P value
Pathological type					
Serous	90	246.88 ± 48.12	0.06	244.82 ± 44.54	0.356
Mucinous	7	192.31 ± 43.64		186.29 ± 59.14	
Clear cell	21	199.83 ± 57.52		208.89 ± 48.28	
Endometrioid	9	239.98 ± 40.04		236.12 ± 71.95	
FIGO stage					
I–II	44	200.93 ± 54.42	0.007	202.55 ± 56.26	< 0.001
III–IV	83	253.98 ± 41.30		252.01 ± 38.24	
Lymphatic metastasis					
No	80	224.83 ± 41.34	0.002	222.98 ± 55.20	0.001
Yes	47	253.95 ± 38.06		255.12 ± 34.60	
Relapse					
Yes	61	263.89 ± 31.91	< 0.001	249.53 ± 42.76	0.002
No	66	209.46 ± 54.51		221.33 ± 54.27	
DOD					
Yes	43	266.52 ± 33.34	< 0.001	260.43 ± 32.87	< 0.001
No	84	219.78 ± 53.70		221.79 ± 53.60	

Italic values indicate statistical significance (P < 0.05)

*HDAC3* Histone deacetylase 3, *FOXA1* Fox protein A1, *DOD* die of disease

In Kaplan–Meier method, FIGO III-IV disease, lymphatic metastasis, HDAC3 high expression and FOXA1 high expression sighificantly decreased patients’ DFS in the univariate analysis (p < 0.001, = 0.001, < 0.001 and = 0.001, respectively; Table 3). FIGO III-IV disease, HDAC3 high expression and FOXA1 high expression sighificantly decreased patients’ OS in the univariate analysis (p = 0.002, < 0.001, and = 0.006, respectively; Table 3). Multivariate analysis, FIGO III-IV disease and HDAC3 high expression were identified as the independent risk factors for recurrence (HR: 3.675 [95% CI 1.728–7.813], p < 0.001 and HR: 2.123 [95% CI 1.188–2.795], p = 0.008, respectively; Table 3). HDAC3 high expression was independently associated with patients’ survival in the multivariate analysis (HR: 4.182 [95% CI 2.003–8.731], p < 0.001, Table 3).

**Table 3:** The survival analysis of the effect of clinical pathological characteristics on prognosis in ovarian carcinoma

Parameter	Relapse		P value ^a^	P value ^b^		DOD		P value ^c^	P value ^d^
	+	−				+	−		
Age									
< 57	24	28	0.172		–	17	41	0.240	–
≥ 57	37	26				26	41		
FIGO staging system									
I–II	9	34	< 0.001		< 0.001	6	37	0.002	0.1
III–IV	52	20				37	45		
Lymphatic metastasis									
No	31	43	0.001		0.595	23	53	0.169	–
Yes	29	10				20	27		
HDAC3 H-score									
Low expreesion	19	40	< 0.001		0.008	9	52	< 0.001	< 0.001
High expression	42	14				34	30		
FOXA1 H-score									
Low expression	23	35	0.001		0.853	14	46	0.006	0.951
High expression	38	19				29	36		

*DOD*  death due to disease, *cm* centimeter, *LNM* lymph node metastasis

^a^single factor analysis of clinical pathological characteristics and disease-free survival

^b^multifactor analysis of clinical pathological characteristics and disease-free survival

^c^single factor analysis of clinical pathological characteristics and overall survival

^d^multifactor analysis of clinical pathological characteristics and overall survival

### HDAC3 and FOXA1 expression levels in human OC cell lines

**Fig. 2:**
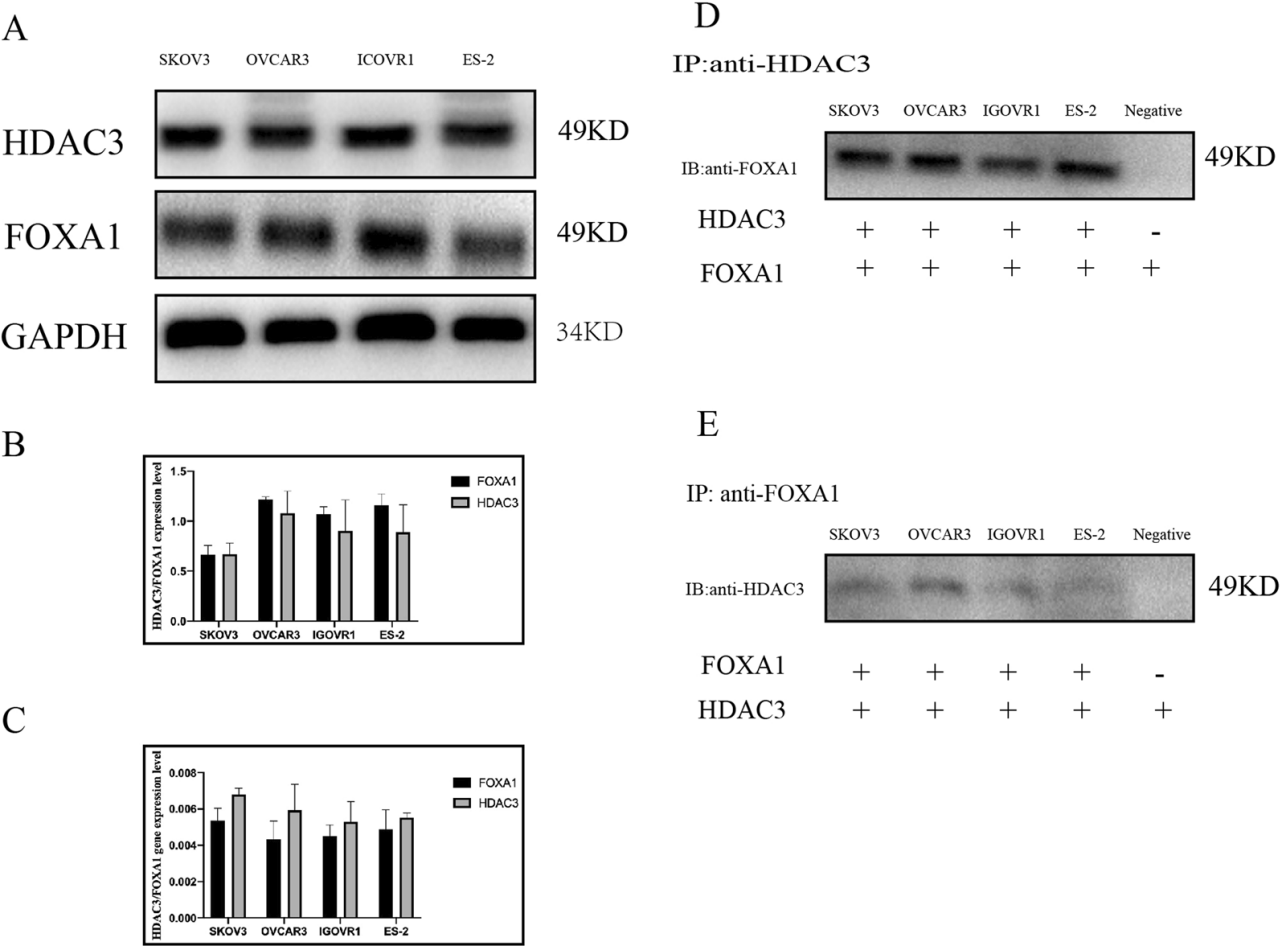
The expression levels of HDAC3 and FOXA1 in human OC cell lines. **A**, **B** Western blot analysis results show the expression levels of HDAC3 and FOXA1 in different human OC cell lines. There was no significant difference in the expression levels of HDAC3 and FOXA1 in the different OC cell lines. **C** RT-PCR results show the expression levels of HDAC3 and FOXA1 in the different types of OC cell lines. There were no differences in the expression profiles of HDAC3 and FOXA1 in the OC cell lines. **D** Immunoprecipitation (IP) of the HDAC3/FOXA1 complex by an anti-HDAC3 antibody and subsequent western blot analysis with anti-FOXA1 antibody. **E** Immunoprecipitation (IP) of the HDAC3/FOXA1 complex by an anti-FOXA1 antibody and subsequent western blot analysis with anti-HDAC3 antibody

HDAC3 and FOXA1 expression levels in four ovarian epithelial cell lines (SKOV3, OVCAR3, IGOVR1 and ES-2) were confirmed by western blot assay and RT-PCR analysis (Fig. 2A–C). Specific antibodies against HDAC3 and FOXA1 were added to the four ovarian epithelial cell lysates, and the results are shown in Fig. 2D, E. The results demonstrated that HDAC3 and FOXA1 established a complex and were able to be immunoprecipitated by either anti-HDAC3 or anti-FOXA1 antibodies. Then, the immunoprecipitated complexes were able to be identified by western blot with anti-HDAC3 (Fig. 2D) or anti-FOXA1 (Fig. 2E) antibodies, respectively.

### Roles of FOXA1 in biological functions in vitro

To further investigate the function of FOXA1 in OC cell proliferation capacity, apoptosis capacity and invasion capacity, SKOV3 and ES-2 cells were transfected with lentiviral vectors.

**Fig. 3:**
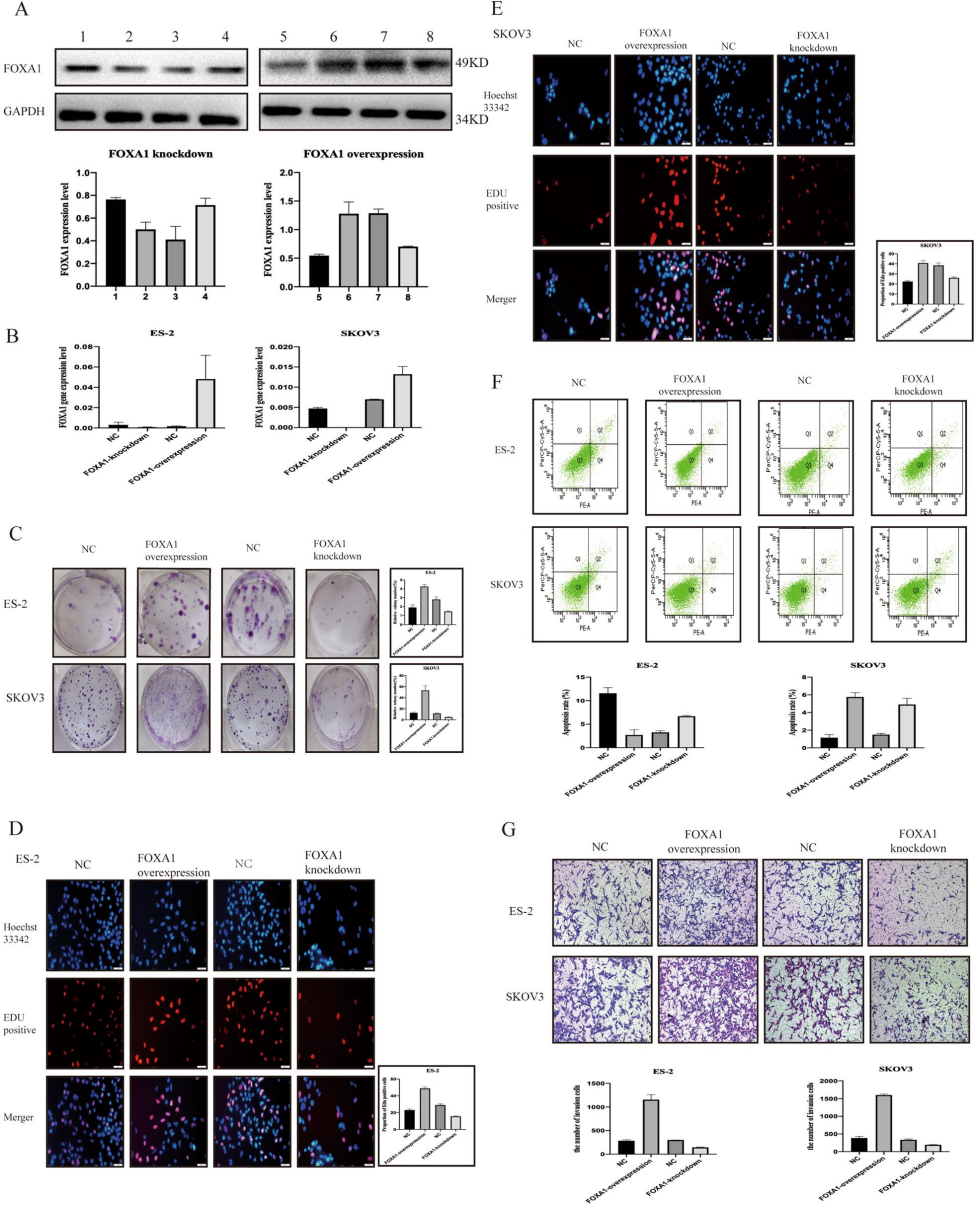
Effects of FOXA1 on OC cell proliferation capacity, apoptosis capacity and invasion capacity. **A** Immunoblot results show the expression of FOXA1 in ovarian cancer cells after FOXA1 transfection. Lanes 1 and 4: mock groups in ES-2 and SKOV3 cells; lanes 2 and 3: FOXA1 low expression groups in ES-2 and SKOV3 cells; lanes 5 and 8: mock groups in ES-2 and SKOV3 cells; lanes 6 and 7: FOXA1 high expression groups in ES-2 and SKOV3 cells. Quantitative data are expressed as FOXA1 relative to GAPDH. **B** RT-PCR results show that FOXA1 expression was increased in the FOXA1 overexpression group and decreased in the FOXA1 knockdown expression group in ES-2 and SKOV3 cells. **C** Cell colony forming results show that overexpression of FOXA1 significantly improved cell colony formation in ES-2 and SKOV3 cells, which was consistent with the EdU results (**D**, **E**). **F** Apoptosis assay results show that FOXA1 overexpression reduced OC cell apoptosis and that FOXA1 knockdown increased OC cell apoptosis. **G** Significantly greater invasion was observed in the FOXA1 overexpression group

RT-qPCR and western blot analysis were used to confirm the transfection efficiency (Fig. 3A, B).

To explore the relationship between FOXA1 and OC proliferation capacity, cell colony forming experiments and EdU assays were performed in SKOV3 and ES-2 cells after FOXA1 transfection. In the cell colony forming experiment (Fig. 3C), the colony forming ability of ES-2/SKOV3 cells in the FOXA1 knockdown group was significantly reduced (p < 0.05), and the colony forming ability of ES-2/SKOV3 cells in the FOXA1 overexpression group was significantly increased (p < 0.05). Furthermore, the EdU proliferation assay demonstrated similar results: there were fewer EDU-positive cells in the FOXA1-knockdown group and more EDU-positive cells in the FOXA1-overexpression group (Fig. 3D, E).

An apoptosis assay was performed to identify the relationship between FOXA1 and OC. By increasing the expression level of FOXA1, flow cytometry assays showed that the apoptosis rates in ES-2 and SKOV3 cells were significantly reduced. In contrast, FOXA1 knockdown significantly enhanced the apoptosis rate in ES-2 and SKOV3 cells (Fig. 3F).

In addition, Transwell assays were performed to identify the association between FOXA1 and OC invasion capacity in SKOV3 and ES-2 cells. After 16 h of incubation and staining, positive cells were counted. Cell invasion capacity was enhanced by increasing the expression level of FOXA1, and invasion capacity was inhibited by decreasing the expression level of FOXA1 (Fig. 3G).

### Correlations of HDAC3 expression with FOXA1 expression and the Wnt/β-catenin signaling pathway

To identify the correlation between HDAC3 and FOXA1 expression, western blotting was performed.

**Fig. 4:**
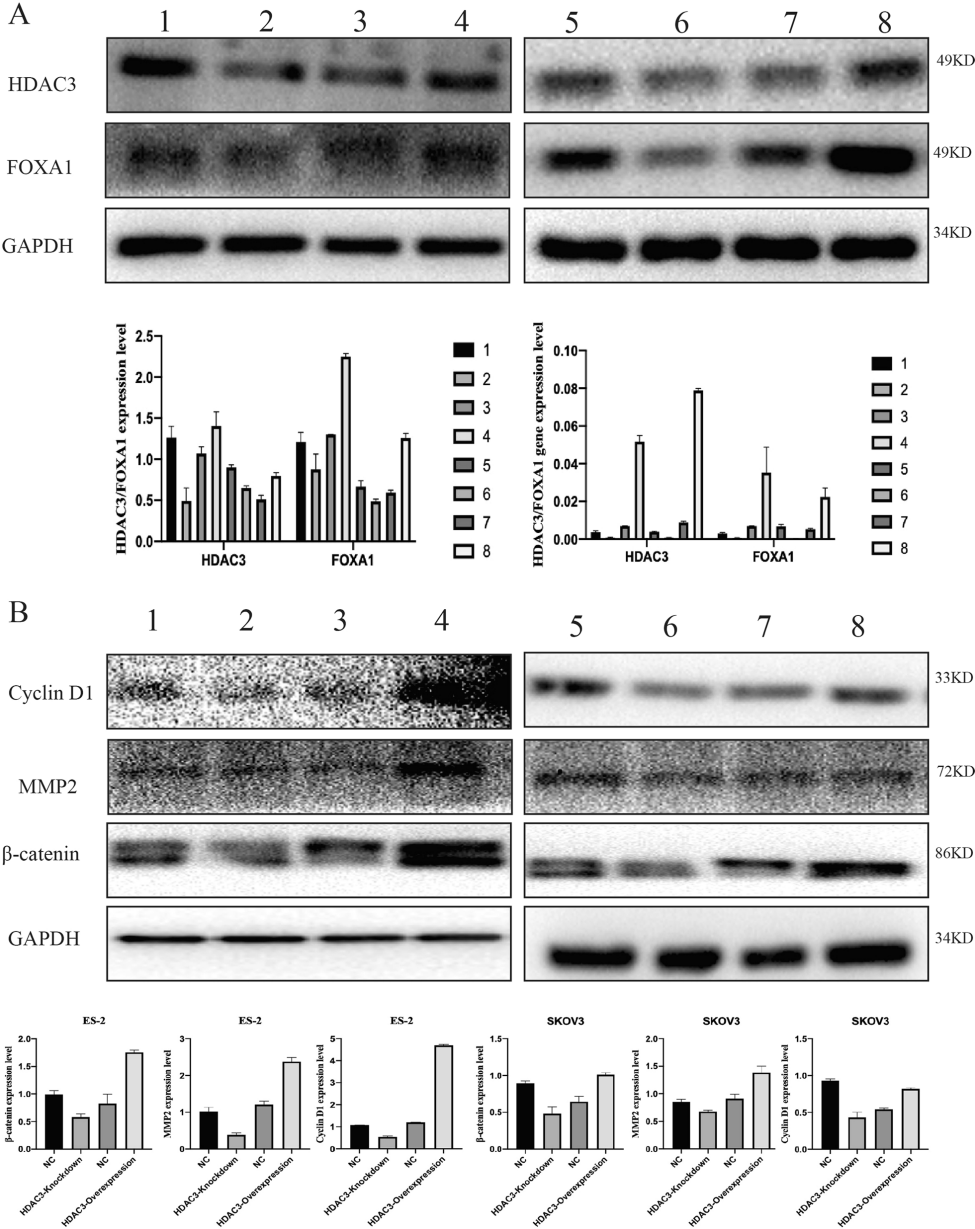
HDAC3 affects FOXA1 and the Wnt/β-catenin signaling pathway. **A** Immunoblot and RT-PCR results show the expression of HDAC3 and FXOA1 in ovarian cancer cells after HDAC3 transfection. Lanes 1 and 5: mock groups in ES-2 and SKOV3 cells; lanes 2 and 6: HDAC3 high expression groups in ES-2 and SKOV3 cells; lanes 3 and 7: mock groups in ES-2 and SKOV3 cells; lanes 4 and 8: HDAC3 high expression groups in ES-2 and SKOV3 cells. Quantitative data are expressed as FOXA1 and HDAC3 relative to GAPDH. **B** Immunoblot results show the expression levels of β-catenin, cyclin D1, MMP2 and GAPDH in ES-2 and SKOV3 cells after HDAC3 transfection. Lanes 1 and 5, mock groups in ES-2 and SKOV3 cells; lane 2 and line 6, HDAC3 low expression group in ES-2 and SKOV3 cells; lanes 3 and 7, mock groups in ES-2 and SKOV3 cells; lane 4 and lane 8, HDAC3 high expression group. Quantitative data are expressed as β-catenin, cyclin D1 and MMP2 relative to GAPDH

RT-qPCR and western blot analysis were used to identify the transfection efficiency after transfecting HDAC3 lentiviral vectors into SKOV3 and ES-2 cells (Fig. 4A). Western blot assays demonstrated significantly decreased levels of FOXA1 in HDAC3-knockdown cells and significantly increased levels in HDAC3-overexpressing cells.

β-catenin, cyclin D1, MMP2 showed decreased levels in the HDAC3 knockdown cells (ES-2: β-catenin/cyclin D1/MMP2, P = 0.0254/0.0046/0.0181, respectively; SKOV3: β-catenin/Cyclin D1/MMP2, P = 0.0284/0.0112/0.0373, respectively) and significantly increased levels in the HDAC3 overexpression cells (ES-2: β-catenin/cyclin D1/MMP2, P = 0.0172/ < 0.0001/0.0082, respectively; SKOV3: β-catenin/Cyclin D1/MMP2, P = 0.0217/0.0030/0.0409, respectively) compared with the corresponding control groups (Fig. 4B).

### Role of HDAC3 and FOXA1 in biological functions in vivo

HDAC3 expression modulated tumor formation in xenograft experiments. Four groups of nude mice were injected with ES-2-HDAC3-knockdown-NC, ES-2-HDAC3-knockdown, ES-2-HDAC3-overexpression-NC, and ES-2-HDAC3-overexpression cells. After a 20-day observation period, the tumor formation rates were 100%.

**Fig. 5:**
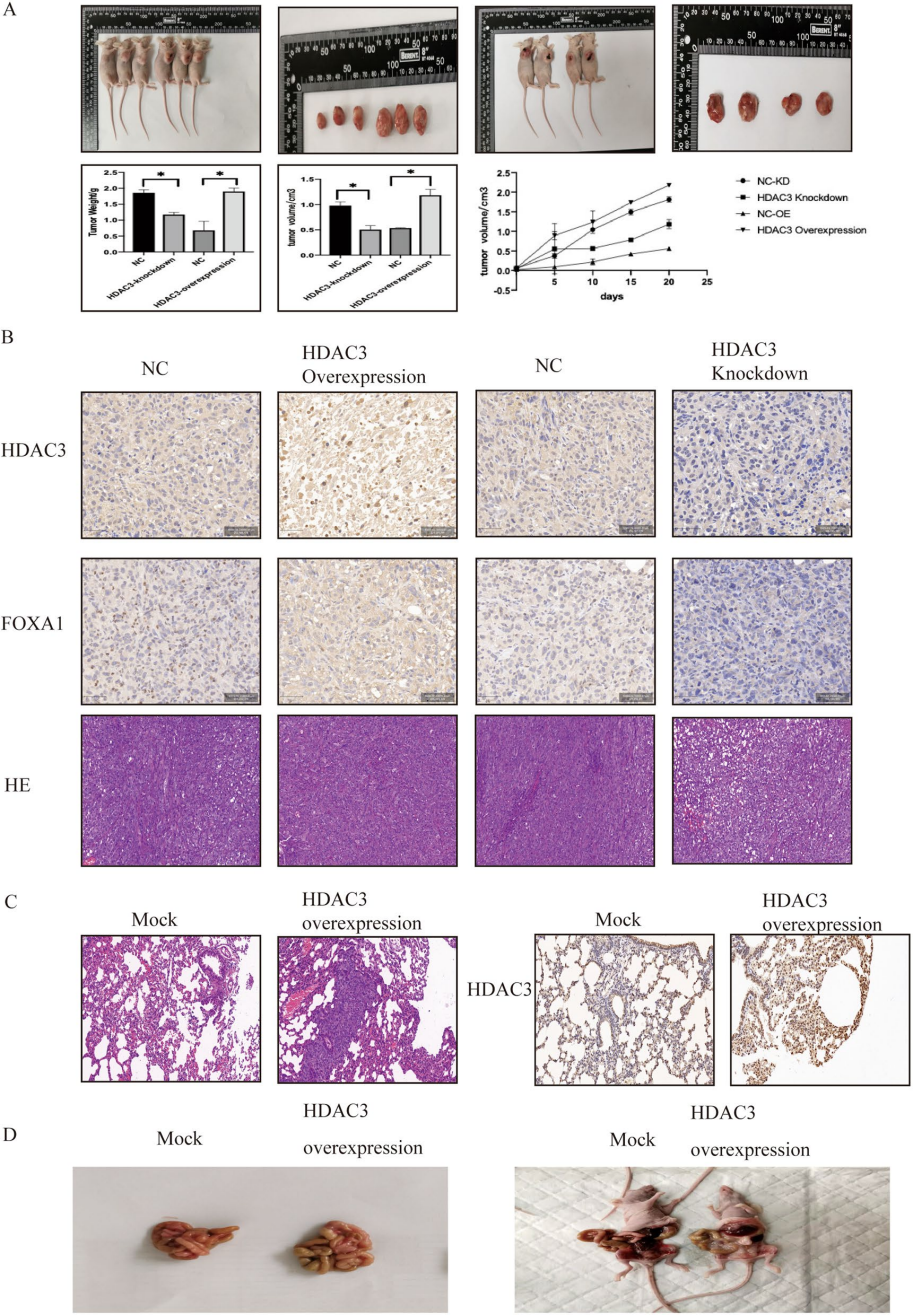
Role of HDAC3 and FOXA1 in biological functions in vivo. **A** Xenograft experiments were performed to test and verify the role of HDAC3 and FOXA1 in tumor formation. The tumor curve is shown. Compared with the NC groups, the tumors in the HDAC3 overexpression group were significantly larger and heavier. In contrast, the tumors in the HDAC3 knockdown group were significantly smaller and lighter. **B** The tumor tissues were fixed in formalin, embedded in paraffin, and stained with H&E. IHC was performed to detect HDAC3 and FOXA1 expression. **C** H&E and IHC staining in lung tissues of xenografts. **D** Representative images of the peritoneal metastasis results

As shown in Fig. 5A, compared with the corresponding control groups, the volumes and weights of tumors in the ES-2-HDAC3-knockdown group were significantly smaller (volumes: P < 0.05 and weights: P < 0.001). In contrast, the volumes and weights of tumors in the ES-2-HDAC3-knockdown group were significantly larger than those in the corresponding control group (volumes: P < 0.05 and weights: P < 0.001). Tumor tissues were fixed with formalin, embedded in paraffin, and stained with H&E and IHC to detect HDAC3 and FOXA1 expression (Fig. 5B).

The metastasis of ovarian cancer was identified in vivo. H&E and immunohistochemical staining showed higher metastasis rates in the overexpression group and lower metastasis rates in the knockdown group (Fig. 5C). Autopsies revealed that peritoneal metastasis occurred in the HDAC3 overexpression group, whereas peritoneal metastasis occurred less often in the mock group than in the HDAC3 overexpression group (Fig. 5D).

## Discussion

OC is the 7th most common cancer and 8th most common cause of cancer death among women, and the five-survival rate is below 45% [2]. More than 90% of OC is EOC in patients over age 40 [2]. Moreover, it is difficult to diagnose EOC early, and resistance to chemotherapy drugs makes the prognosis of EOC poor [26]. Recently, surgical skills have improved, and the application of platinum-based combined chemotherapy has improved OC prognosis, but the long-term survival rate is still unsatisfactory [27]. Early diagnosis and treatment of ovarian cancer can decrease its mortality rate. In recent cancer research, more and more biochemical and molecular markers were discovered. In these studies, molecular markers have been used in cancer diagnosis, prognosis and treatment. Muhammad Tarek Abdel Ghafar et al. [28] found that HtrA2 can improve the diagnosis accuracy of breast cancer, and be related with prognosis of patients. Early diagnosis helps improve the patients of breast cancer prognosis. In chronic myeloid leukemia, micro-RNA-150 can predict the efficacy of imatinib treatment, and found the miR-1501 expression on day 14 of imatinib treatment is a useful early predictor for imatinib response [29]. It can help to choose a better treatment way and evaluate the effect of treatment. Serum angiogenetic markere neuropilin-1 (NRP-1) combinated with AFP can enhance the diagnostic power in hepatocellular carcinoma, and predicted the poor prognosis [30]. We want to find a molecular marker for early diagnosis of ovarian cancer. Our study aimed to identify the molecular mechanism of EOC to understand the pathogenesis of EOC and find a better treatment.

The FOX protein family can be divided into 17 subclasses according to the amino acid sequence of the forkhead domain [31]. The FOX protein family is involved in developmental disease, metabolic disease and tumors [32]. FOXA1, one member of the FOX protein family, interacts with cis-regulatory regions in heterochromatin to enhance the interaction of ERs with chromatin [33]. FOXA1 is also involved in pancreatic cancer, lung cancer, liver cancer and other solid tumors by regulating AR and ER signaling pathways [34]. EOC is also a hormone-dependent disease, and thus we wanted to explore the relationship between FOXA1 and EOC. In our research, we identified that the expression of FOXA1 was increased in EOC and that the expression level of FOXA1 was significantly enhanced in FIGO stages III-IV. In addition, the high expression of FOXA1 influenced the prognosis of patients with EOC. FOXA1 is associated with the malignant biological behavior of tumors. In pancreatic cancer, overexpression of FOXA1 promotes the invasion ability by affecting the progression of EMT [35]. FOXA1 also plays an important role in promoting cell proliferation and suppressing apoptosis in hepatocellular carcinoma [36]. In gastric carcinoma, FOXA1 promotes tumor cell proliferation, migration and invasion ability [37]. Similar to our study, FOXA1 promoted EOC cell proliferation, migration and invasion but decreased apoptosis. HDAC3 expression is upregulated in gastric cancer, while knocked down expression levels of HDAC3 have been shown to reduce gastric cancer cell proliferation [5]. HDAC3 was identified as a promoting factor of EOC in our previous in vitro study [10]. In this study, we discovered a regulatory relationship between HDAC3 and FOXA1. In the assessment of the TMAs, HDAC3 was also significantly increased in FIGO stage III-IV and could influence the prognosis of patients with EOC. More importantly, there was a positive correlation between HDAC3 expression and FOXA1 expression in EOC tissues. Both the in vitro and in vivo improvement of the expression level of HDAC3 increased the expression of FOXA1, and knockdown of the expression level of HDAC3 decreased the expression of FOXA1.

The Wnt/β-catenin signaling pathway plays an important role in the progression of various tumors. HDAC3 has a close relationship with the Wnt/β-catenin signaling pathway. In colon cancer, HDAC3 knockdown can suppress β-catenin translocation from the plasma membrane to the nucleus and increase the expression of Wnt inhibitors TLE1, TLE4 and SMO [38]. Our results indicated that HDAC3 also altered the Wnt/β-catenin signaling pathway in EOC and regulated the expression level of FOXA1.

The number of patients was small in our study. Some bioinformation analysis of data download from cancer databases regarding FOXA1 and HDAC3 expression should be added in the further analysis. And a larger number of patients are recommended to support our results. The relationship between HDAC3 and FOXA1 requires further study, and it is necessary to determine whether other molecules participate in the function of HDAC3 and FOXA1 in OC.

## Conclusions

**Fig. 6:**
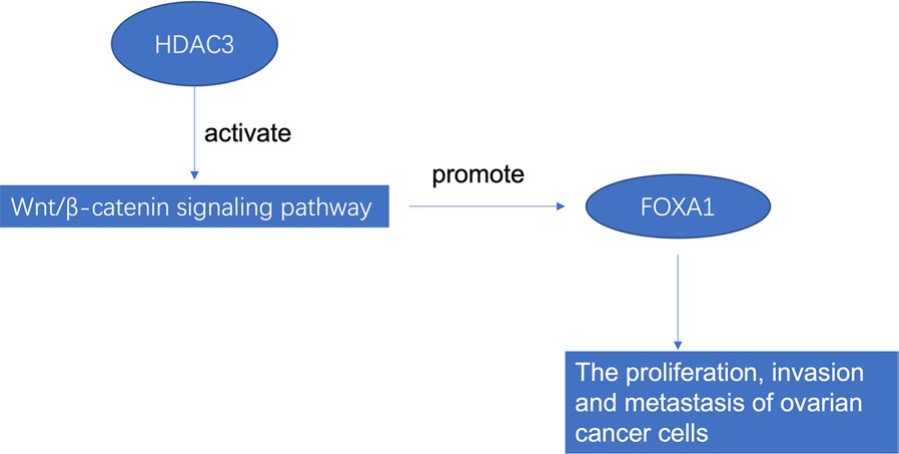
A schematic diagram illustrating the role of FOXA1 in relation to HDAC3 and Wnt/β-catenin signaling pathway in promoting EOC progression and metastasis

Patients with higher expression levels of FOXA1 and HDAC3 protein have a poor prognosis than those with low expression levels. FOXA1 plays essential roles in the proliferation, apoptosis and invasion of OEC cell lines and in xenograft experiments, with HDAC3 modulating FOXA1 expression possibly through the activity of the Wnt/β-catenin signaling pathway (Fig. 6).

## Data Availability

Due to the nature of this research, participants of this study did not agree for their data to be shared publicly, so supporting data is not available.
